# First‐line pembrolizumab for non–small cell lung cancer patients with PD‐L1 ≥50% in a multicenter real‐life cohort: The PEMBREIZH study

**DOI:** 10.1002/cam4.2806

**Published:** 2020-02-05

**Authors:** Karim Amrane, Margaux Geier, Romain Corre, Hervé Léna, Guillaume Léveiller, Florence Gadby, Régine Lamy, Jean‐Louis Bizec, Eric Goarant, Gilles Robinet, Sylvie Gouva, Gilles Quere, Ronan Abgral, Ulrike Schick, Cyril Bernier, Christos Chouaid, Renaud Descourt

**Affiliations:** ^1^ Oncology Department CHRU Brest Brest France; ^2^ Pulmonology Department CHU Hôpital Ponchaillou Rennes France; ^3^ Pulmonology Department CH Yves le Foll Saint Brieuc France; ^4^ Pulmonology Department CH des Pays Morlaix Morlaix France; ^5^ Oncology Department CH Bretagne Sud Lorient France; ^6^ Pulmonology Department CH Bretagne Atlantique Vannes France; ^7^ Pulmonology Department CH Saint‐Malo Saint Malo France; ^8^ Nuclear Medicine Department CHRU Brest Brest France; ^9^ Pulmonology Department CH René Pleven Dinan France; ^10^ Pulmonology Department CHI Créteil Créteil France

**Keywords:** first‐line monotherapy, non–small cell lung cancer, PD‐L1 tumor proportion score, pembrolizumab, Real world

## Abstract

**Background:**

The KEYNOTE‐024 trial demonstrated that pembrolizumab, a PD‐1 inhibitor, significantly improves progression‐free survival (PFS) and overall survival (OS) in selected patients with previously untreated advanced non–small cell lung cancer (NSCLC) with a PD‐L1 tumor proportion score (TPS) ≥50% and without EGFR/ALK aberrations. The main aim of this study was to report the efficacy and safety profile of pembrolizumab in real‐life conditions.

**Method:**

This was a French retrospective multicenter longitudinal study of 108 consecutive patients with advanced NSCLC, a PD‐L1 TPS ≥50% and without EGFR/ALK aberrations who were treated by pembrolizumab, in first line. Patient data were obtained from medical files.

**Results:**

The main characteristics of the cohort were: median age [range] 66.7 [37‐87] years, 64.8% male, 23.1% with a performance status (PS) of 2, and 88.9% current or former smokers. Eighty‐seven percent had stage IV NSCLC at diagnosis, 9.2% untreated brain metastases at inclusion,. With a median follow‐up of 8.2 months, the median PFS was 10.1 months (95% CI, 8.8‐11.4). The objective response rate was 57.3% (complete response 2.7%, partial response 54.6%). Disease control rate was 71.1%. At 6 months, the OS rate estimated was 86.2%. Treatment‐related adverse events (AE) of grade 3 occurred in 8% of patients. There were no grade 4 or 5 AEs.

**Conclusion:**

In a real‐life cohort of advanced NSCLC patients (including PS 2 and untreated brain metastases), with PD‐L1 TPS ≥50%, pembrolizumab demonstrates similar PFS to the pivotal clinical trial.

## Graphical abstract

This retrospective multicenter longitudinal study was intended to report the efficacy and safety profile of pembrolizumab in real‐life conditions for the first‐line treatment of advanced squamous none small‐cell lung cancer. The median progression free survival was 10.1 months (95% CI, 8.8‐11.4) and the treatment‐related adverse events of grade 3 occurred in 8% of patients. This study demonstrates similar progression‐free survival to KEYNOTE‐024.

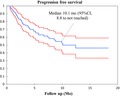

## 1 INTRODUCTION

Non–small cell lung cancer (NSCLC) is a common and deadly malignancy with over 2.1 millions new diagnoses and over 1.8 million associated deaths per year worldwide.^1^ In 2018, according to the World Health Organization (WHO), lung cancer ranked first in terms of mortality in man, with 1.2 million estimated deaths per year, and fourth in terms of incidence, with 2.1 million new cases estimated, (28% of all cancers). While the incidence of lung cancer is on the decrease in men, it continues to increase in women from 3.6% in 1980 to 19.2% today because of an inversion in smoking habits.

In the past, patients with advanced (unresectable or metastatic) NSCLC had a median survival of 8‐12 months.^2^, ^3^ Fortunately, over the last 10 years, we have seen an appreciable increase in effective therapies that are significantly improving clinical outcomes, raising the hope of a substantial decrease in NSCLC‐associated mortality.^4^, ^5^, ^6^

These therapeutic advances are broadly represented by two approaches: genetic‐ and immune‐based therapies. Genetic‐based therapies comprise treatments that address a common driver mutations (Epithelioma Growth Factor Receptor = EGFR mutation) seen in 11% of NSCLC tumors and ALK (Anaplastic lymphoma Kinase) translocations in 5%.^7^ The EGFR inhibitors have received approval from the results of randomized trials demonstrating a benefit on improved progression‐free survival (PFS) and objective response rate (ORR).^8^, ^9^, ^10^, ^11^, ^12^ New immunotherapies, known as immune checkpoint inhibitors, have also played an integral role in advancing outcomes for patients with advanced NSCLC in recent years. These drugs are monoclonal antibodies that bind to and interfere with negative regulator receptors on T lymphocytes, which in turn allow the lymphocytes to remain active and target NSCLC cells.^13^, ^14^ The programmed death‐1 (PD‐1) inhibitors, such as nivolumab and pembrolizumab, are humanized monoclonal antibodies that block the PD‐1 receptor, a negative regulator on lymphocytes. These agents have a more favorable tolerance profile compared to that of chemotherapy for improving results and improve the results.^4^, ^5^, ^6^ Pembrolizumab was approved for advanced NSCLC first‐line with PD‐L1 Tumor Proportion Score (TPS) ≥50% based on the results of KEYNOTE‐024. This phase III randomized trial showed a median PFS of 0.3 months (95% CI, 6.7 to NR) for immunotherapy versus 6.0 months (95% CI, 4.2‐6.2) for chemotherapy,, with a hazard ratio (HR) for disease progression or death of 0.50 (95% CI, 0.37‐0.68); *P* < .001).^4^ Median OS was 30.0 months (95% CI, 18.3‐NR) in the pembrolizumab arm versus 14.2 months (95% CI, 9.8‐19.0) in the chemotherapy group with an HR of 0.63 (95% CI, 0.47‐0.86; *P* = .002).^15^ Another randomized trial KEYNOTE‐042 investigated pembrolizumab in the NSCLC first‐line treatment with PD‐L1 score ≥1%. In the subgroup of patients with a PD‐L1 ≥50%, median PFS was 7.1 months (95% CI, 5.9‐9.0) in the pembrolizumab group versus 6.4 months (95% CI, 6.1‐6.9) in the chemotherapy group with an HR for disease progression or death of 0.69 (95% CI, 0.56‐0.85; *P* = .0003).^16^

These important results support the benefit of pembrolizumab first line for treating advanced NSCLC. However, there is little data about its use in routine clinical practice in terms of safety and efficacy outside the clinical trial setting. The purpose of this study was to investigate the real‐world utilization of pembrolizumab in patients with advanced NSCLC.

## 2 MATERIALS AND METHODS

### 2.1 Study design and patients

This noninterventional, retrospective study was conducted in nine centers of the Brittany region in France. Patients were identified by each investigator from a database. To be eligible, patients had to have a histologically proven NSCLC or, if not in cytology, advanced (III) or metastatic (IV) stage with a PD‐L1 ≥50%, a WHO performance status (PS) of 0, 1 or 2 and without organ dysfunction. All patients were treated with pembrolizumab in first line. Exclusion criteria were previous EGFR or ALK aberrations, past medical history of active hepatitis (B or C virus) or positive test of human immunodeficiency virus (HIV) or a severe, uncontrolled autoimmune disease. Centers were asked to include all eligible patients consecutively over the period of inclusion (June 2017 through December 2018).

### 2.2 Data collection

The patients’ data were obtained retrospectively from medical files and included demographics, characteristics of NSCLC, number and localization of metastatic sites, tumor response to pembrolizumab, treatments after progression and toxicities. Histology, the PD‐L1 score and genetic abnormalities were evaluated locally in each center.

PD‐L1 TPS was obtained by commercially available assays: PD‐L1 IHC 22C3 clone (68.1%),^17^ QR1 clone (16.7%),^18^ E1L3N clone (5.5%),^19^ and for 9.2% clone was not specified.

### 2.3 Ethical considerations

The study was conducted in accordance with the Declaration of Helsinki and was approved by the French Advisory Committee on Information Processing in Health Research (CCTIRS). If patients were alive, they received a written information on the study. For deceased patients, an exemption of information was obtained from CCTIRS.

### 2.4 Study measures

PFS was the primary endpoint, defined as the time from initiation of pembrolizumab to the date of disease progression or death. Secondary endpoints included OS, defined as the time from randomization to death from any cause and ORR, defined as the percentage of patients with a confirmed complete or partial response, according to RECIST (version 1.1), and safety. Adverse events (AE) graded by each participating center according to the National Cancer Institute Common Terminology Criteria for Adverse Events (CTACE), version 4.0.

Tumors were assessed locally, and the radiology reports reviewed by the trial coordinating investigators.

### 2.5 Statistical analysis

This is a descriptive study for which no theoretical calculation of the number of patients to be included has been made. Both PFS and OS were estimated by the Kaplan‐Meir method. Age, sex, histology (squamous vs non‐squamous), brain metastases at baseline, mutations, and stage were applied to the stratified log‐rank.

XLSTAT‐Life software (Addinsoft), was used for statistical analysis. 0.05 was significance level of *P*‐values.

## 3 RESULTS

### 3.1 Characteristics of patients at diagnosis of NSCLC

**Figure 1:**
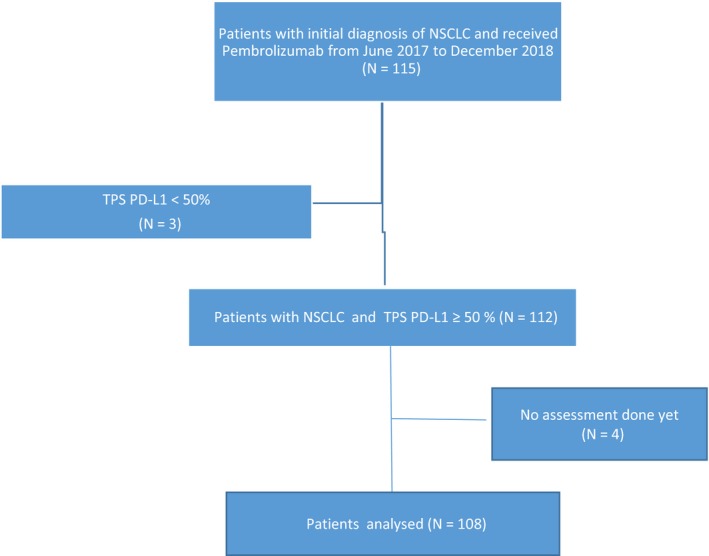
Flow chart of study profile

We identified 115 patients with advanced NSCLC who received pembrolizumab from June 2017 through December 2018. Three patients were excluded because of a TPS <50% and four patients who had not been assessed. Finally, 108 were included in the analysis (Figure 1).

Patients’ main characteristics are described in Table 1. Overall, 23.1% of the patients had a PS of 2 and 17.6% had brain metastases at the inclusion.

**Table 1:** Characteristics of patients at baseline

Characteristics	No of patients (n = 108)
Age, y; median (range)	67 (37‐87)
Sex (male/ female)	70/38
ECOG performance status, n (%)	
0	17 (15.8)
1	53 (49.1)
2	25 (23.1)
Unknown	13 (12.0)
Smoking status, n (%)	
Current	34 (31.5)
Former	62 (57.4)
Never	4 (3.7)
Unknown	8 (7.4)
Histology, n (%)	
Squamous	28 (25.9)
Non squamous	80 (74.1)
Mutations, n (%)	
KRAS	18 (16.6)
BRAF	4 (3.7)
MET	3 (2.7)
ROS1	1 (0.9)
Brain metastasis, n (%)	
Yes	19 (17.6)
No	89 (82.4)
Stage, n (%)	
III	14 (13.0)
IV	94 (87.0)

The date of primary data cut off was February 07, 2019. At this date, 102 patients had a follow‐up of at least 6 months. Median periods of follow‐up was 8.2 months (range, 0.9‐ 20.9); median time to treatment was and 7.3 months (range, 0.6 to not reached).

### 3.2 Efficacy

**Figure 2:**
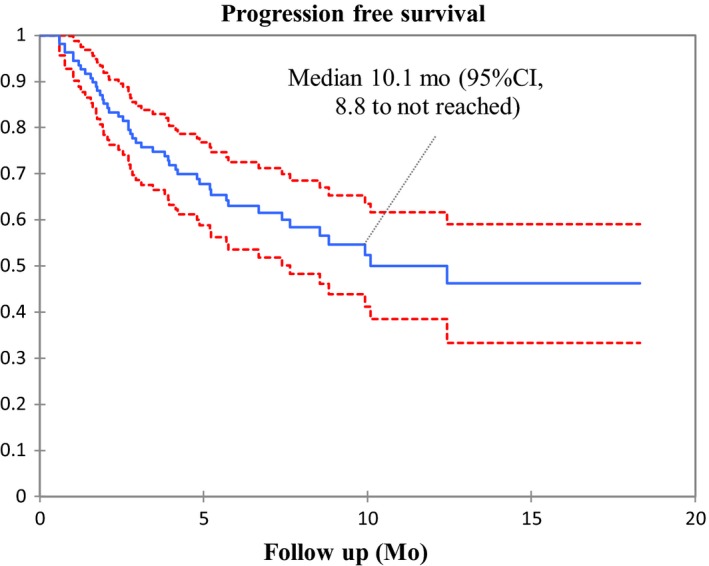
Kaplan‐Meier estimates of progression‐free survival

**Figure 3:**
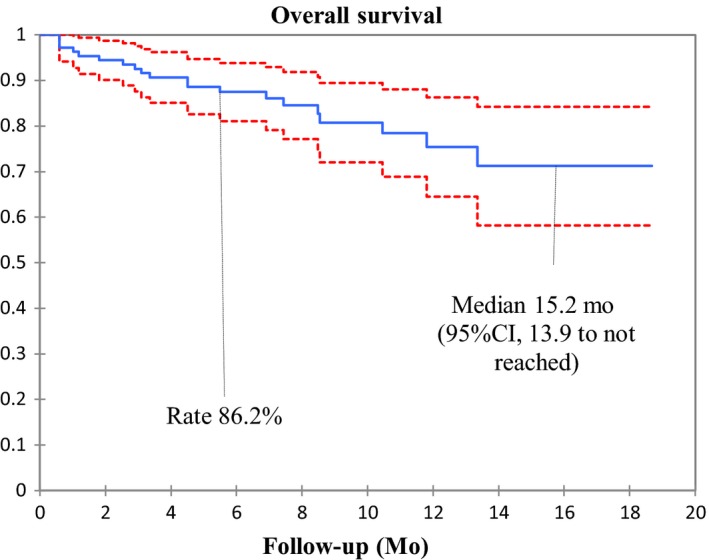
Kaplan‐Meier estimates of overall survival (95% confidence interval in red)

Median PFS and OS were 10.1 months (95% CI, 8.8 to 11.4) (Figure 2) and 15.2 months (95% CI, 13.9 to not reached), respectively (Figure 3). At 6 months, the estimated percentages of patients who had no disease progression and were alive were, respectively, 62.7% and 86.2%. On the basis of 20 total events of death, median OS was 15.2 months (95% CI, 13.9 to not reached) (Figure 3).

The estimated 6‐month OS rate was 86.2%.

No parameter tested in univariate analysis was significant for PFS (Table 2). Analysis for OS was not performed due to lack of events.

**Table 2:** Univariate analysis of clinical factors with PFS before the beginning of treatment

Parameter	Median PFS in months (95% CI)	*P* value
Age (y)		.208
<65	8.3 (6.7‐9.9)	
≥65	10.9 (9.1‐12.6)	
ECOG score		.412
PS 0‐1	10.4 (8.9‐11.9)	
PS ≥2	6.8 (5.0‐8.6)	
Sex		.878
Male	10.3 (8.6‐11.9)	
Female	8.4 (6.9‐9.9)	
Smoking status		.903
Current	10.8 (8.4‐13.3)	
Former	9.7 (8.1‐11.3)	
Never	3.9 (3.5‐4.4)	
Histology		.381
Squamous	9.0 (6.5‐11.5)	
Non‐squamous	10.6 (9.0‐12.1)	
Brain metastasis		.288
Yes	10.9 (7.8‐13.9)	
No	9.5 (8.1‐11.0)	
Mutations		.910
Yes	9.8 (7.4‐12.3)	
No	10.0 (8.4‐11.5)	
Stage		.827
III	9.7 (6.6‐12.7)	
IV	9.9 (8.5‐11.3)	

Of the 108 evaluable patients, objective responses were observed in 62 patients (ORR = 57.4%), with three complete (2.7%) and 59 partial responses (54.6%). Fifteen patients (13.8%) had a stable disease and 31 (28.7%) a progressive disease. The median delay of response was 1.9 months (range, 0.9‐3.2 months).

### 3.3 Adverse events

During treatment, AEs occurred in 46.3% of the patients and AE data was missing for 10.2%. Grade 3 AEs occurred in 6 (8%) patients (essentially renal and skin reaction). No grade 4 or 5 AEs were reported. Four (3.7%) patients discontinued treatment due to treatment‐related AEs (Table 3).

**Table 3:** Treatment‐related AEs

AEs	Grade 1‐2, n (%)	Grade 3, n (%)
Diarrhea/colitis	12 (16)	–
Pneumonitis	1 (1.3)	–
Pruritus/rash	22 (29.3)	2 (2.6)
Hypo/hyperthyroidism	20 (26.6)	–
Renal toxicity	2 (2.6)	2 (2.6)
Neurologic/muscular toxicity	9 (12)	2 (2.6)
Adrenal insufficiency	1 (1.3)	–
Anemia	2 (2.6)	–

The median delay between the first administration of pembrolizumab and occurrence of a clinicobiological AE was 11 (95% CI, 3‐55).weeks.

## 4 DISCUSSION

This real‐world retrospective observational study evaluating pembrolizumab as first‐line treatment for advanced NSCLC patients with a TPS ≥50% and without sensitizing EGFR mutations or ALK translocations, showed a PFS of 10.1 months (95% CI, 8.8 to not reached). In univariate analysis, PFS was not associated with any prespecified clinical factors. At the time of data analysis, pembrolizumab was associated with a high rate of OS: only 13.7% of the enrolled patients had died. We also observed a high ORR (57.4%), and a low frequency of treatment‐related AEs.

Our PFS findings are consistent with those of pembrolizumab in the pivotal phase 3 KEYNOTE‐024 trial, albeit with a shorter follow‐up: The median PFS in our study of 10.1 months was almost identical to that in KEYNOTE‐024 (10.3 months).^4^, ^20^ However, our study was a retrospective analysis based on a nonclinically selected population including 23% of patients with PS 2. This is worth noting as patients with PS 2 may respond differently to immune checkpoint inhibitors and have poorer survival rates than patients with PS 0 or 1. At the same time, PS 2 patients represent a heterogeneous population.^21^ On the contrary, the patients enrolled in KEYNOTE‐024 were highly selected: out of a total of 1653 patients whose tumors could be evaluated for TPS, 500 patients were ≥50% but only 300 of these were included in the study. One explanation for our similar PFS rates could be that more patients had squamous histology in our cohort than in KEYNOTE‐024 (25.9% vs 18.8%) and squamous cell carcinoma seems to be associated with longer PFS on immunotherapy.^22^, ^23^, ^24^ Another explanation could be the trial design. Ours was a retrospective work based on assessment by local investigators without centralized review. Nevertheless, the ORR in our study was concordant with that of KEYNOTE‐024 (57.3% vs 44.3%). It should be noted that results with first‐ line pembrolizumab in TPS ≥50% NSCLC vary from one study to another. KEYNOTE‐042 compares pembrolizumab to chemotherapy for previously untreated NSCLC with TPS ≥1%. PFS was 7.1 months (95% CI 5.9‐9.0) for the subgroup of patients with TPS ≥50%,^16^ far from the 10.3 months of KEYNOTE‐024. No explanations for the difference in PFS were given by the authors, except the heterogeneity of KEYNOTE‐042 population (Table 4).

**Table 4:** Main results of PEMBREIZH, KEYNOTE‐024 and KEYNOTE‐042

Parameter	PEMBREIZH	KEYNOTE‐024^4^, ^20^	KEYNOTE‐042 (TPS ≥50% subgroup)^16^
PFS	10,1 mo 95% CI [8,8 to NA]	10.3 mo 95% CI [6,7 to NA]	7,1 mo 95% CI [5,9 to 9,0]
OS	15,2 mo 95% CI [13,9 to NA]	30,0 mo 95% CI [18,3 to NA]	20,0 mo 95% CI [15,4 to 24,9]
ORR	53,7%	44,8%	39%
IrAEs *	8%	9,7%	8%

Abbreviations: IrAEs: immune‐related adverse events; ORR: Objective response rate; OS: Overall survival; PFS: Progression‐free survival

The OS rate in our study differed to that of KEYNOTE‐024 4 after a follow up of 6 months (86.2%.vs 80.2%), potentially due to a confounding effect of immature data).

In this analysis, the rate of any grade AE was low 46.3%, compared to the rate reported on KEYNOTE‐024 and KEYNOTE‐042 (73.4% vs 63%, respectively), As was the treatment discontinuation rate due to AEs, 3.7% in this analysis compare to vs 7.1% and 9% in the pivotal studies. However, it is difficult to compare our results with those of KEYNOTE 024 and KEYNOTE‐042 as retrospective data collection can underestimation the rate of AE (Table 4).

No patient in our study died from an AE compare to one patient in KEYNOTE‐024 and 13 patients (2%) in KEYNOTE‐042. This suggests effective management of AEs in routine clinical practice.

The results of KEYNOTE‐024 changed the landscape for advanced NSCLC patients with TPS ≥50%. Monoimmunotherapy with pembrolizumab is now the standard of care in first‐line treatment. However, results from recent phase 3 trials evaluating combinations of immunotherapy and chemotherapy will probably further change therapeutic strategy. Among these, the KEYNOTE‐189 trial compared the combination of pembrolizumab or placebo with platinum pemetrexed in first‐line treatment of patients with nonsquamous advanced NSCLC. Co‐primary endpoints (PFS and OS) were positive. After a median follow‐up of 10.5 months, the estimated rate of OS at 12 months was 69.2% (95% CI, 64.1‐73.8) in the pembrolizumab‐chemotherapy combination group versus 49.4% (95% CI, 42.1‐56.2) in the placebo‐chemotherapy combination group (HR for death, 0.49; 95% CI, 0.38‐0.64; *P* < .001). Another trial, KEYNOTE‐407 randomized patients with squamous advanced NSCLC into two arms: pembrolizumab in combination with chemotherapy (carboplatin‐paclitaxel) or placebo combined with the same regimen. After a median follow‐up of 7.8 months, the median OS (primary endpoint) was 15.9 months (95% CI, 13.2 to not reached) in the pembrolizumab‐chemotherapy combination group versus 11.3 months (95% CI, 9.5‐14.8) in the placebo‐chemotherapy combination group (HR for death, 0.64; 95% CI, 0.49‐0.85; *P* < .001). The median PFS was 6.4 months (95% CI, 6.2‐8.3) in the pembrolizumab‐combination group and 4.8 months (95% CI, 4.3‐5.7) in the placebo‐combination group (HR for disease progression or death, 0.56; 95% CI, 0.45‐0.70; *P* < .001). The important point in those trials is the improved OS which was consistent regardless of the TPS and this benefit was higher in patients with TPS ≥50%. In view of these promising new results, the place of pembrolizumab monotherapy in future therapeutic strategies will probably be under discussion. Direct comparison between pembrolizumab and pembrolizumab‐chemotherapy in NSCLC with TPS ≥50% has never been evaluated in a phase 3 trial. Differences in the safety profile could help us to resolve this question but data about AEs in pembrolizumab‐chemotherapy combination trials like KEYNOTE‐189 or KEYNOTE‐407 are not available for the subgroup of patients with TPS ≥50%.

Furthermore, questions remain concerning the TPS cut‐off of 50%. Jiminez Aguilar et al^25^ compared according to TPS, PFS, OS and ORR in patients with NSCLC which received pemrolizumab in first‐line treatement and found that higher TPS levels (of 75%‐100%) were associated with improved clinical outcomes compared to patients with a TPS of 50%‐74%. This result may guide the therapeutic decision between a pembrolizumab‐chemotherapy combination and monotherapy although the fact remains that there is no data about this comparison. Finally, health‐related quality‐of‐life is improved with pembrolizumab than with chemotherapy^26^ and is more cost‐effective,^27^, ^28^ but there are no health‐related QOL data for pembrolizumab‐chemotherapy even if the combination would appears to be cost‐effective.^29^

The results of this retrospective observational study should be considered in the context of the strengths and limitations of the data source and study design. Limitations include the ethnicity of our cohort which was reported as being entirely Caucasian. Although we cannot exclude the possibility of omissions in data entry and errors in physician‐reported outcomes. Secondly, this study suffered from a heterogeneous radiological response assessment to treatment. The time before first evaluation is not homogeneous, and in some cases, this evaluation is carried out by FDG PET/CT,.^30^ Thirdly, not all the AEs were graded strictly according CTCAE 4.0. Nonetheless, the existence of unrecognized confounders is always possible in observational studies. Baseline data were incomplete for some patients, and the severity of AEs and the primary reason for treatment discontinuation were not always correctly cataloged. Finally, the number of events is relatively low, and should be confirmed with a higher number of patients.

However; this multicenter real‐world study to assess pembrolizumab in SSCLC patients with a TPS ≥50% reflects the French routine practices. Our findings provide only a snapshot of a narrow window of time in a very rapidly evolving field of therapy, with limited follow‐up for some patients even though we allowed for a minimum of 12 months of follow‐up for all patients.

## 5 CONCLUSION

In a real‐world French cohort of advanced NSCLC patients (including patients with PS 2 and untreated brain metastases) with TPS ≥50%, pembrolizumab demonstrates a similar PFS to the KEYNOTE‐024 phase 3 trial and a lower rate of AEs. These findings support the effectiveness and safety of pembrolizumab in this setting.

## CONFLICT OF INTEREST

All authors declare that they have no conflict of interest.

## ETHICS APPROVAL AND CONSENT TO PARTICIPATE

Patients gave written informed consent to participate in the study and to have their medical records released. Our institution's local Ethics Committee approved the study design, then the study was registered on clinicaltrials.gov. (Clinicaltrials.gov number not yet assigned).

## Data Availability

The datasets generated and/or analysed during the current study are not publicly available but are available from the corresponding author on reasonable request.
